# TRIM14 promotes colorectal cancer cell migration and invasion through the SPHK1/STAT3 pathway

**DOI:** 10.1186/s12935-018-0701-1

**Published:** 2018-12-11

**Authors:** Zhonghai Jin, Hongguang Li, Xiaofei Hong, Guangrong Ying, Xiaofeng Lu, Lilei Zhuang, Shenbao Wu

**Affiliations:** Department of Gastroenterology, Yiwu Hospital, Wenzhou Medical University, 699 Jiangdong Middle Road, Yiwu, 322000 China

**Keywords:** TRIM14, STAT3, SPHK1, Migration, Invasion

## Abstract

**Background:**

Colorectal cancer (CRC) is one of the most lethal malignancies. Tripartite Motif Containing 14 (TRIM14) is a member of TRIM family proteins, which are involved in the pathogenesis of various cancers. This study aimed to investigate TRIM14 expression in CRC tissues, and its effects on the migration and invasion of CRC cell lines.

**Methods:**

TRIM14 mRNA expression was detected by real-time PCR analysis. Cell migration and invasion were measured by Transwell assays. Protein expression was assessed by western blot analysis.

**Results:**

The expression of TRIM14 was significantly higher in CRC tissues than in matched non-cancerous tissues. TRIM14 knockdown by specific short hairpin RNA (shRNA) attenuated CRC cell migration and invasion, whereas TRIM14 overexpression caused reverse effect. Moreover, TRIM14 positively regulated the protein levels of sphingosine kinase 1 (SPHK1) and phosphorylated STAT3 (p-STAT3), as well as the mRNA and protein expression of matrix metalloproteinase 2, MMP9 and vascular endothelial growth factor, which are transcriptional targets of the STAT3 signaling pathway. Importantly, the blockage of the SPHK1/STAT3 signaling pathway by SKI-II or AG490 could reverse the TRIM14-promoted CRC cell migration and invasion.

**Conclusions:**

Our results reveal a critical role for TRIM14 in promoting migration and invasion of CRC cells, and suggest TRIM14 may serve as a potential molecular target to prevent CRC metastasis.

**Electronic supplementary material:**

The online version of this article (10.1186/s12935-018-0701-1) contains supplementary material, which is available to authorized users.

## Background

Colorectal cancer (CRC) is one of the most common causes of cancer-related death in many countries. The main reason of high mortality rate in CRC patients is the extreme difficulty in treating distant metastases [1]. The feature characteristics of aggressive metastatic cancers include high ability of migration, consequent invasion and adhesion in the distant organs [2]. Until now, the complex mechanisms that lead to cancer metastasis are far from being fully understood.

Members of the tripartite motif (TRIM) family, also called the RING B-box-coiled-coil (RBCC) family, are involved in the pathogenesis of various cancers by acting as oncogenes or tumor suppressor genes [3]. A recent study has reported that TRIM14 expression was down-regulated in non-small cell lung cancer (NSCLC) and played a tumor suppressive role in NSCLC progression [4]. Conversely, studies showed that TRIM14 expression was up-regulated in hepatocellular carcinoma (HCC) [5], osteosarcoma [6], oral squamous cell carcinoma (OSCC) [7], tongue squamous cell carcinoma (TSCC) [8], breast cancer [9] and glioma [10], and that TRIM14 overexpression promoted cell proliferation, migration, invasion and chemoresistance, supporting the oncogenic role of TRIM14 in these cancers. However, few reports have focused on the expression and functions of TRIM14 in CRC.

STAT3 belongs to the signal transducer and activator of transcription (STAT) family, which is activated by the upstream stimuli, such as cytokines, growth factors and non-receptor tyrosine kinases. In response to these stimuli, STAT3 is phosphorylated at Tyr 705, which induces the dimerization of STAT3 through phosphotyrosine-SH2 domain interaction [11, 12]. The dimerized STAT3 translocates to the nucleus, interacts with specific DNA elements and then stimulates the transcription of target genes [11, 12]. STAT3 is found constitutively activated in many human cancers, which is related to malignant characteristics, including rapid proliferation, migration, invasion and metastasis [11, 13]. The critical role of the STAT3 signaling pathway in CRC progression also has been reported [14]. Sphingosine kinase 1 (SPHK1) catalyzes the formation of sphingosine 1-phosphate (S1P), which promotes tumor growth, angiogenesis and metastasis [15]. SPHK1 is found to be upregulated in CRC, and associated with CRC progression and prognosis [16]. A recent study has reported that SPHK1 leads to the constitutive activation of STAT3 in colitis-associated CRC [17]. TRIM8, another member of TRIM proteins, was found to enhance the STAT3 signaling pathway in various cell types [18–20]. TRIM14 overexpression increased the phosphorylation of STAT3 in breast cancer cells [9], while it was unclear whether TRIM14 was related to the STAT3 signaling during CRC progression.

In the present study, TRIM14 expression was elevated in CRC tissues. Knockdown of TRIM14 repressed CRC cell migration and invasion as well as the levels of phospho-STAT3 (p-STAT3), matrix metalloproteinase 2 (MMP2), MMP9 and vascular endothelial growth factor (VEGF). Importantly, inhibition of STAT3 signaling attenuated TRIM14-enhanced cell invasion and migration of CRC cells. Our findings indicate that TRIM14 represents a novel therapeutic target in CRC.

## Materials and methods

### Patient samples

The study was approved by the Ethics Committee of Yiwu Hospital, Wenzhou Medical University (Yiwu, China). A total of 40 CRC patients (24 men and 16 women, age range: 33–71 year) who underwent surgical resection at Department of Gastroenterology, Yiwu Hospital were enrolled in this study after written informed consent were obtained from all the patients. During surgery, CRC and matched non-cancerous colorectal tissue samples were obtained. Part of the specimens were subjected to hematoxylin and eosin (HE) staining for histopathological analysis, and the other part of the specimens were immediately snap-frozen and stored at − 80 °C. The histopathological stage of each CRC was determined by the pathologists according to the tumor-node metastasis (TNM) classification system. Clinical characteristics, including gender, age, tumor size and tumor stage, were retrieved from the medical records (Table 1).

**Table 1:** Clinicopathological characteristics (n = 40)

Characteristic	Cases	%
Gender		
Male	24	60.0
Female	16	40.0
Age (years)		
≥65	26	65.0
<65	14	35.0
Tumor size (cm)		
≥ 5.0	21	52.5
< 5.0	19	47.5
Clinical stage		
I/II	18	45.0
III	22	55.0

### Real-time PCR analysis

The Trizol reagent (Invitrogen Carlsbad, CA, USA) was used to isolate total RNA from the tissue samples and cell lines per the manufacturer’s protocol. Complementary DNA (cDNA) was then synthesized from the isolated total RNA with cDNA synthesis kit (Thermo Fisher, Rockford, IL, USA), and used as template in Real-time PCR analysis on an ABI 7300 system (Applied Biosystem, Foster City, CA, USA) with SYBR Green qPCR Master Mixes (Thermo Fisher). The oligonucleotides used as PCR primers are listed in Table 2. Gene expression was normalized to the expression of GAPDH by 2^−ΔΔCT^ methods [21].

**Table 2:** Primer pairs used for real-time PCR

Gene	Primers (forward/reverse)
TRIM14	5′-GGATTTGTGTCTCCGTTCTG-3′ and 5′-TCTGTCTGCCTGGTATTCTG-3′
VEGF	5′-ATTTCTGGGATTCCTGTAG-3′ and 5′-CAGTGAAGACACCAATAAC-3′
MMP2	5′-TACAGGATCATTGGCTACACACC-3′ and 5′-GGTCACATCGCTCCAGACT-3′
MMP9	5′-TGTACCGCTATGGTTACACTCG-3′ and 5′-GGCAGGGACAGTTGCTTCT-3′
GAPDH	5′-CACCCACTCCTCCACCTTTG-3′ and 5′-CCACCACCCTGTTGCTGTAG-3′

### Cell lines and culture conditions

Human CRC cell lines SW620, Caco2, LoVo, HT-29 and SW1116 obtained from Shanghai Institute of Biochemistry and Cell Biology (SIBCB, Shanghai, China) were grown at 37 °C in a humidified atmosphere containing 5% CO_2_ and 95% air. All cell lines were cultured in RPMI-1640 (Hyclone, Rockford, IL, USA) plus 10% fetal bovine serum (FBS, Hyclone) and 100 units/ml penicillin and streptomycin.

### RNA interference and overexpression of TRIM14

TRIM14 short hairpin RNAs (shTRIM14) or control shRNA (shNC) was constructed in PLKO.1 vector (Addgene, Cambridge, MA, USA). The cDNA encoding full-length human TRIM14 was constructed in PLVX-puro Vector (Clontech, Palo Alto, CA, USA). The constructs were confirmed by sequencing. Lentiviruses were produced in 293T cells by contransfecting lentiviral constructs with packaging plasmids for 48–72 h. The collected viral supernatant was filtered through 0.45-μm filter, and infected target cell lines in the presence of polybrene (8 μg/ml, Sigma, St. Louis, MO, USA). The shRNA sequence against TRIM14 are shown as follows: shTRIM14#1: CAGATTACTACTTGACGAA; shTRIM14#2: CATTGGACATTCGCCTTAA; shTRIM14#3: GATCGCTATTGCTGAAATA.

### Transwell migration and invasion assays

Transwell migration assays were performed using Transwell plates with 8 μm pore filters (Costar, Manassas, VA, USA). Viral transduced cells were serum-starved overnight. Cells suspended in 0.2 ml serum-free medium were plated into the upper chamber of Transwell plates (5 × 10^4^ per well), and 0.6 ml medium with 10% FBS were loaded to the lower chamber. After incubation at 37  °C for 24 h, cells in the upper chamber were completely removed with a cotton swab, while cells that migrated to the lower chamber were fixed in 4% paraformaldehyde and stained with 0.1% crystal violet. Then stained cells were counted in five random fields at 200× magnification, and the average number was taken. The average number of control group was set as 100%, and relative migration and invasion of other groups was calculated by comparison of the cell number with the average number of control group. For invasion assays, the experiment procedure was similar to migration assays except that the Transwell insert was coated with Matrigel (BD Biosciences, Franklin Lakes, NJ, USA) before the cells were loaded.

### Western blot analysis

RIPA buffer (Beyotime, Shanghai, China) plus protease inhibitor cocktail (Roche, Indianapolis, IN, USA) was used to lysate cultured cells. Equal amount of protein (30 μg per sample) was separated by SDS–polyacrylamide gel electrophoresis (SDS-PAGE) gel and transferred onto the nitrocellulose membrane. The membranes were blocked and probed at 4 °C overnight with the following primary antibodies: STAT3 (#9139), and GAPDH (#5174) from Cell Signaling Technology (Danvers, MA, USA); SPHK1 (Ab71700), p-STAT3 (Ab76315), VEGFA (Ab51745), MMP2 (Ab37150) and MMP9 (Ab38898) from Abcam (Cambridge, MA, USA). The membranes were then probed with horseradish peroxidase (HRP)-conjugated secondary antibody at room temperature for 1 h. Signal was detected by the enhanced chemiluminescence reagents (Thermo Fisher). GAPDH was used as loading control. Representative blots of three independent experiments are shown.

### Statistical analysis

All analyses were done with GraphPad Prism software (La Jolla, CA, USA). Data are presented as mean ± standard deviation (SD). Significance of statistical analysis was assessed using ANOVA tests. P < 0.05 was considered as statistically significant.

## Results

### Up-regulation of TRIM14 mRNA expression in CRC tissues

**Fig. 1:**
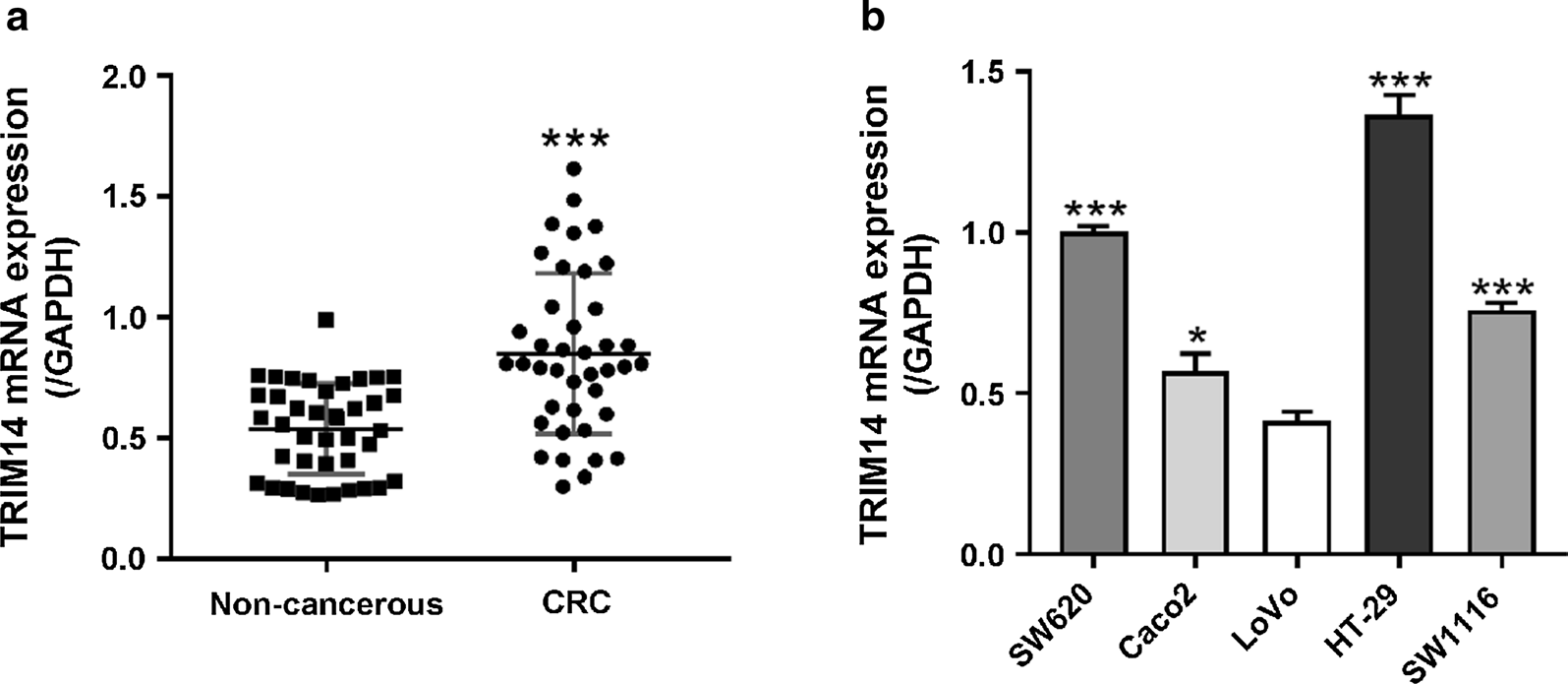
mRNA expression of TRIM14 in human CRC tissues and CRC cell lines. **a** Real-time PCR analysis of TRIM14 expression in 40 pairs of non-cancerous colon and CRC tissues. TRIM14 expression were normalized to the expression of GAPDH. ***P < 0.001. **b** TRIM14 expression in 5 CRC cell lines by real-time PCR analysis. *P < 0.05, ***P < 0.001 versus LoVo cells

We tested the mRNA expression of TRIM14 in CRC tissues and matched non-cancerous tissues by real-time PCR analysis. TRIM14 mRNA levels were markedly up-regulated in CRC tissues (Fig. 1a). Additionally, TRIM14 mRNA was detected in various CRC cell lines including SW620, Caco2, LoVo, HT-29 and SW1116 (Fig. 1b). TRIM14 mRNA was highly expressed in HT-29 and SW620 cell lines, and lowly expressed in LoVo cells.

### TRIM14 promoted CRC cell migration and invasion

**Fig. 2:**
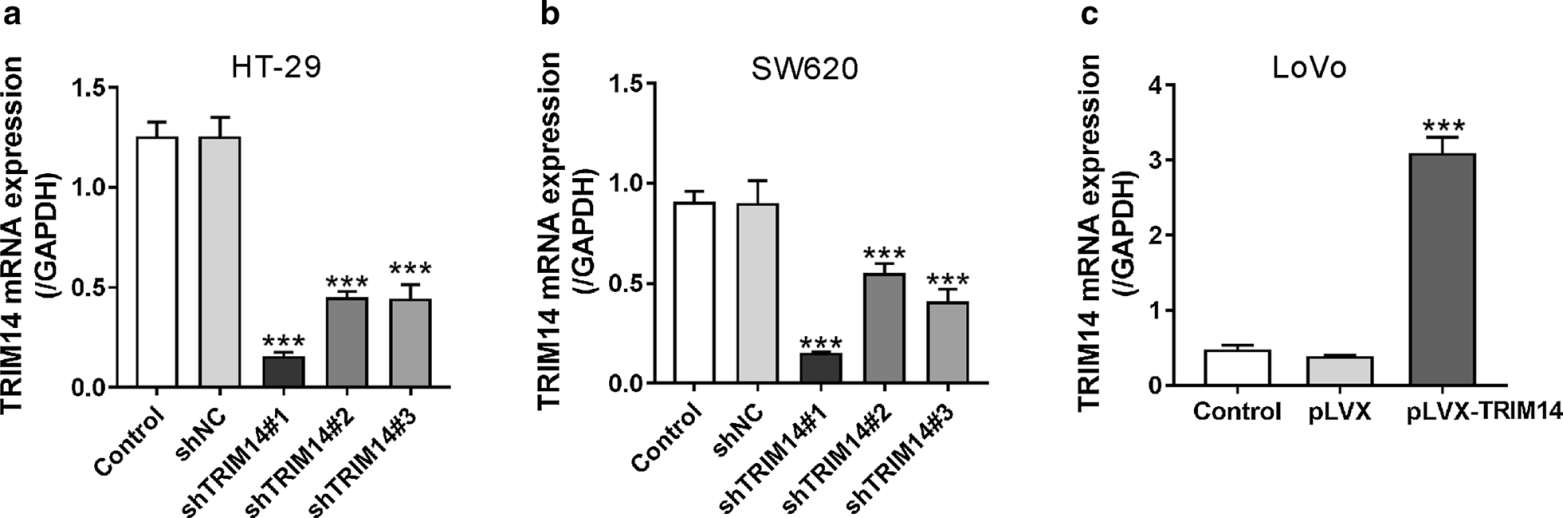
Manipulation of TRIM14 expression in CRC cells. HT-29 (**a**) and SW620 cells (**b**) were transduced with shTRIM14#1, #2, #3 or shNC. LoVo cells (**c**) were transduced with pLVX-TRIM14 or pLVX. Cells without any treatment were served as negative Control. TRIM14 expression was assessed by real-time PCR analysis. ***P < 0.001 versus shNC or pLVX

**Fig. 3:**
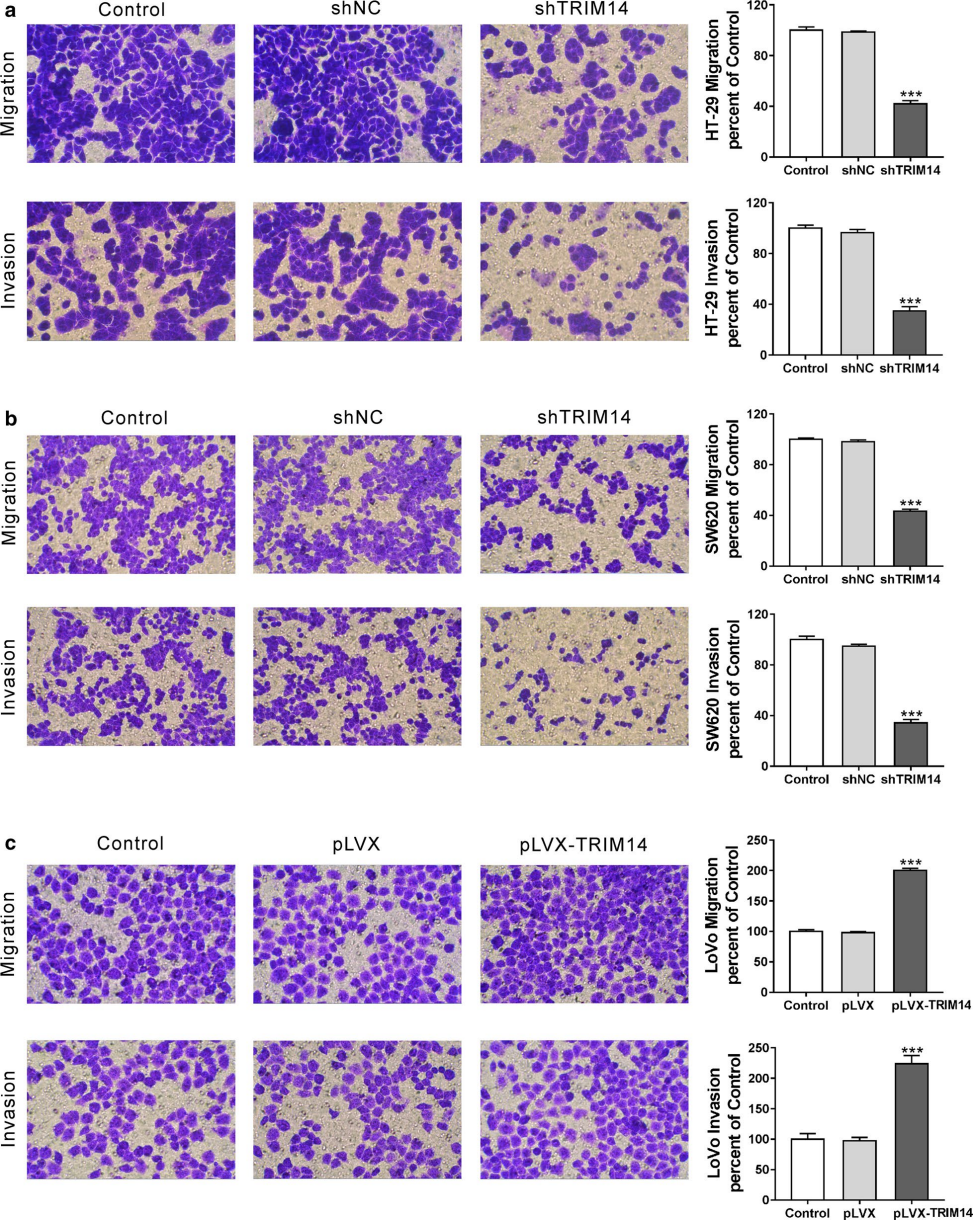
TRIM14 affected the migration and invasion of CRC cells. HT-29 (**a**) and SW620 cells (**b**) were transduced with shTRIM14#1 or shNC. LoVo cells (**c**) were transduced with pLVX-TRIM14 or pLVX. Transwell migration and invasion assays were performed to determine the effect of TRIM14 expression on the migration and invasion of CRC cell lines at 24 h after treatment. Cells without any treatment were served as negative Control. The stained cells were counted in five random fields at ×200 magnification, and the average number was taken. The average number of Control group was set as 100%, and relative migration and invasion of other groups was calculated by comparison of the cell number with the average number of control group. ***P < 0.001 versus shNC or pLVX

We then explored whether TRIM14 expression affected CRC cell migration and invasion. We manipulated TRIM14 expression in CRC cells by lentiviral infection. As indicated in Fig. 2a, b, all the 3 TRIM14 shRNA (shTRIM14#1, #2 and #3) significantly down-regulated TRIM14 expression in both HT-29 and SW620 cells, and shTRIM14#1 was the most efficiency one and used in the subsequent assays. As Fig. 2c shown, pLVX-TRIM14 markedly up-regulated TRIM14 expression in LoVo cells. Inhibition of TRIM14 in HT-29 (Fig. 3a and Additional file 1: Figure S1A) and SW620 cells (Fig. 3b and Additional file 1: Figure S1B) attenuated cell migration and invasion, whereas ectopic expression of TRIM14 in LoVo cells enhanced invasion and migration (Fig. 3c and Additional file 1: Figure S1C) at 12 h and 24 h after plating.

### TRIM14 activated the SPHK1/STAT3 signaling pathway in CRC cells

**Fig. 4:**
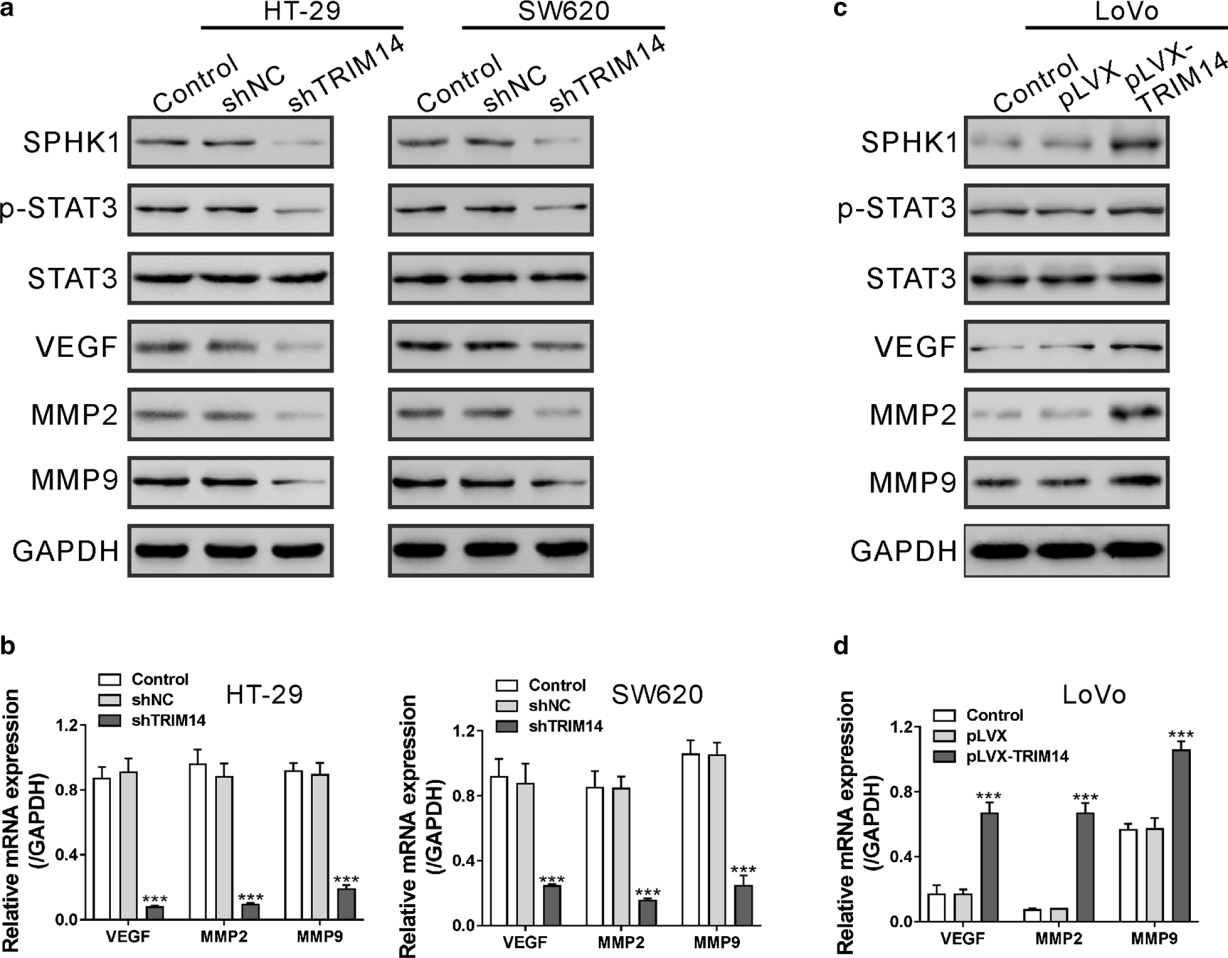
TRIM14 activated the SPHK1/STAT3 signaling pathway in CRC cells. HT-29 and SW620 cells (**a**, **b**) were transduced with shTRIM14#1 or shNC. LoVo cells (**c**, **d**) were transduced with pLVX-TRIM14 or pLVX. The expression of relevant proteins was detected by real-time PCR and western blotting analyses at 48 h post transduction. ***P < 0.001 versus shNC or pLVX

A recent study has shown that TRIM14 enhanced the STAT3 signaling pathway in breast cancer cells [9], which plays a critical role in CRC progression [14]. SPHK1 is upregulated in CRC cancer patients [16] and leads to constituent activation of STAT3 in colitis-associated CRC [17]. Thus, we examined whether TRIM14 affected the SPHK1/STAT3 signaling in CRC cells. TRIM14 knockdown repressed the levels of SPHK1 and phosphorylated STAT3, while they had no effect on the levels of STAT3 (Fig. 4a). TRIM14 knockdown repressed the mRNA and protein levels of MMP2, MMP9 and VEGF (Fig. 4a, b), which are transcriptional targets of the STAT3 signaling pathway [22]. Reverse results were observed in LoVo cells overexpressing TRIM14 (Fig. 4c, d). Collectively, these data suggest that TRIM14 can enhance the SPHK1/STAT3 signaling cascade in CRC cells.

### Inhibition of SPHK1/STAT3 signaling with SKI-II/AG490 counteracted the promontory effects of TRIM14 on CRC cell migration and invasion

**Fig. 5:**
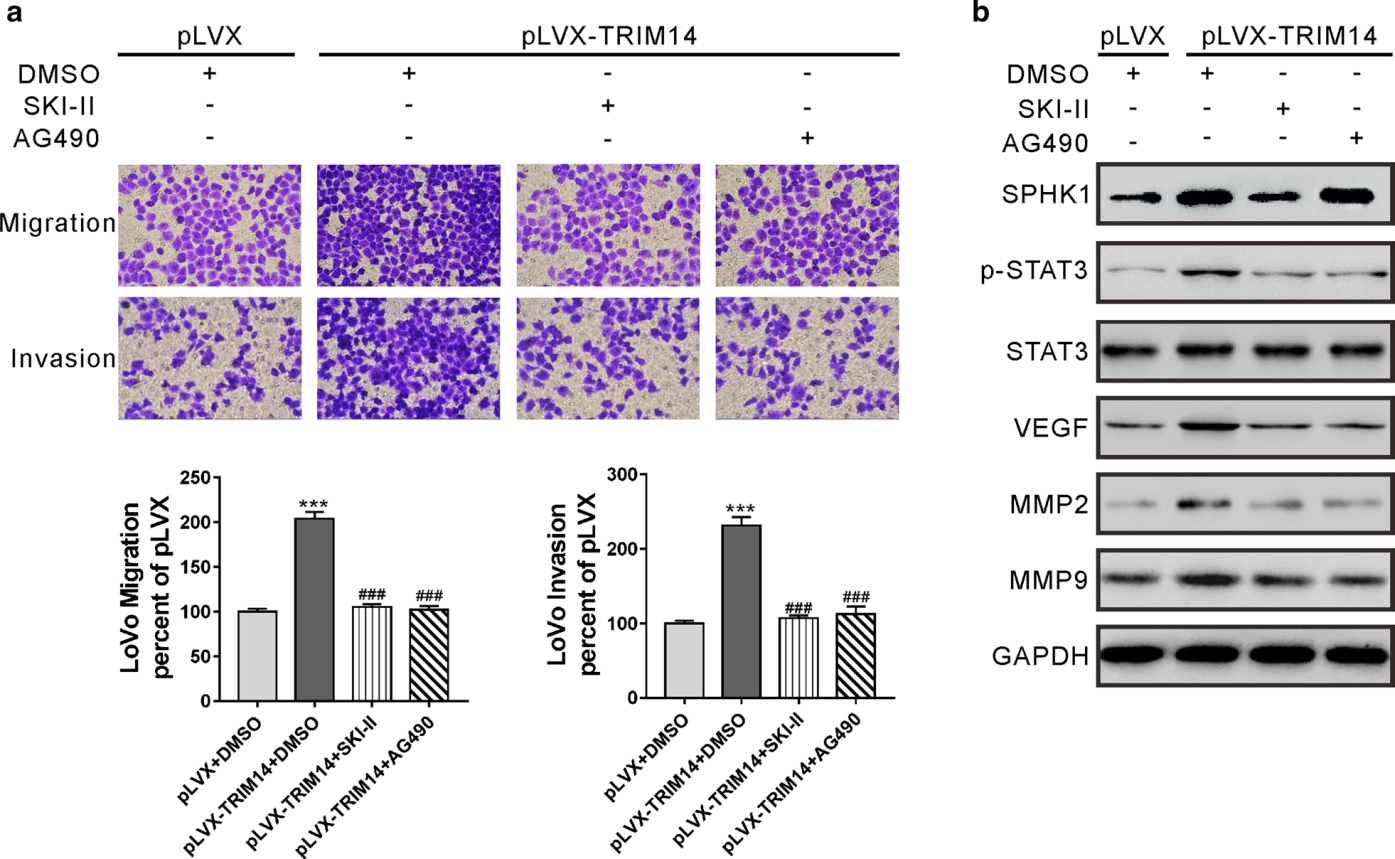
TRIM14 promoted migration and invasion of CRC cells through activating the SPHK1/STAT3 signaling. LoVo cells were transduced with pLVX-TRIM14 or pLVX, and treated with 5 μM SKI-II (Selleck Chemicals, Houston, TX, USA), 30 μM AG490 (Selleck Chemicals) or vehicle (DMSO). **a** Transwell migration and invasion assays were performed. The stained cells were counted in five random fields at ×200 magnification, and the average number was taken. The average number of Control group (cells transduced with pLVX and treated with DMSO) was set as 100%, and relative migration and invasion of other groups was calculated by comparison of the cell number with the average number of control group. ***P < 0.001 versus pLVX + DMSO; ^###^P < 0.001 versus pLVX-p53 + DMSO. **b** The levels of SPHK1, p-STAT3, STAT3, VEGF, MMP2 and MMP9 were detected

To assess whether the effects of TRIM14 on CRC cells were mediated by the SPHK1/STAT3 signaling, LoVo cells were infected with pLVX-TRIM14 virus and treated with a SPHK1 inhibitor SKI-II [23] or a STAT3 inhibitor AG490. As expected, the inhibition of SPHK1 or STAT3 signaling mostly blocked the promontory effects of TRIM14 on migration and invasion (Fig. 5a), as well as the levels of p-STAT3, VEGF, MMP2 and MMP9 (Fig. 5b).

## Discussion

Evidences have shown that TRIM family proteins participate in the pathogenesis of various cancers [3]. For example, TRIM24 [24, 25] and TRIM29 [26, 27] are up-regulated in CRC tissues and exert oncogenic functions on CRC. In the current study, TRIM14 expression was higher in CRC tissues than in paired normal colorectal tissues (Fig. 1). In line with our findings, previous studies have reported the overexpression of TRIM14 in HCC, OSCC, TSCC, osteosarcoma, glioma and breast cancer [5–10]. On the contrary, decreased TRIM14 expression was observed in NSCLC [4]. These results suggest the different expression pattern and function of TRIM14 in diverse cancers.

Distant metastases contributes to the high mortality rate in CRC patients [1]. Tumor metastasis is a multistage process. Tumor cell migration and invasion are responsible for the infiltration of tumor cells into lymphatic vessels, blood vessels and distant organs, and represent for the key steps during tumor metastasis [28, 29]. The function of TRIM14 in cancer cell migration and invasion has been described in osteosarcoma [6] and OSCC [7]. Consistently with the above reports, our data showed that TRIM14 knockdown inhibited CRC cell migration and invasion, while TRIM14 overexpression had opposite effects (Fig. 3). Our data further demonstrated the important role of TRIM14 in tumorigenesis.

Several cancer-related signaling pathways have been reported to be activated by TRIM14, such as AKT pathway in osteosarcoma [6], NF-κB pathway in TSCC [8], Wnt/β-catenin pathway in glioma [10] and STAT3 in breast cancer [9]. The present study focused on the STAT3 pathway, and the effects of TRIM14 on the other pathways will be considered in our future study. STAT3 signaling pathway plays an important role in tumor-related activities, such as cell proliferation, migration, invasion and metastasis [11, 13]. Evidence has shown that STAT3 is constitutively activated in CRC [30], which is critical for CRC cell growth, survival, invasion and migration [14]. It has been demonstrated that SPHK1, which catalyzes the formation of sphingosine 1-phosphate (S1P) [15], causes the persistent activation of STAT3 in colitis-associated CRC [17]. In the present study, TRIM14 overexpression promoted CRC cell migration and invasion, which was counteracted by SKI-II or AG490, suggesting that SPHK1/STAT3 contributed to TRIM14-mediated CRC cell migration and invasion. Matrix metalloproteinases (MMPs) are key enzymes responsible for the degradation of extracellular matrix, thus involving in tumor metastasis [31]. Numerous studies have suggested the increased expression and activity of MMP2 and MMP9 in CRC specimens [32–35]. VEGF, an important signal protein, is associated with the progression, invasion and metastasis of CRC and may be independent prognostic marker for CRC patients [36]. VEGF, MMP2 and MMP9 are known as transcriptional targets of the STAT3 signaling pathway [22]. Here, TRIM14 overexpression increased the phosphorylation of STAT3, and the expression of its downstream targets MMP2, MMP9 and VEGF in CRC cells, further demonstrating the involvement of the SPHK1/STAT3 pathway in the function of TRIM14. TRIM14 contains the PRYSPRY domain, which is crucial for protein–protein interactions [37]. A previous study has identified more than 70 TRIM14 interacting proteins, which are associated with protein ubiquitination [4]. We speculated that TRIM14 may regulate SPHK1 expression via its interaction partner, although the precise mechanism how TRIM14 regulated the SPHK1/STAT3 pathway requires further investigation.

## Conclusions

This study reveals the increased expression of TRIM14 in CRC samples, and an important role of TRIM14 in CRC cell migration and invasion through the SPHK1/STAT3 signaling. TRIM14 may be a potential molecular target to prevent CRC metastasis.

## Additional file

**Additional file 1: Figure S1.** TRIM14 affected the migration and invasion of CRC cells. HT-29 (A) and SW620 cells (B) were transduced with shTRIM14#1 or shNC. LoVo cells (C) were transduced with pLVX-TRIM14 or pLVX. Transwell migration and invasion assays were performed to determine the effect of TRIM14 expression on the migration and invasion of CRC cell lines at 12 h after treatment. Cells without any treatment were served as negative Control. The stained cells were counted in five random fields at 200× magnification, and the average number was taken. The average number of Control group was set as 100%, and relative migration and invasion of other groups was calculated by comparison of the cell number with the average number of control group. ***P < 0.001 versus shNC or pLVX.

## Abbreviations

CRC: colorectal cancer
TRIM: tripartite motif
RBCC: RING B-box-coiled-coil
NSCLC: non-small cell lung cancer
HCC: hepatocellular carcinoma
OSCC: oral squamous cell carcinoma
TSCC: tongue squamous cell carcinoma
STAT: signal transducer and activator of transcription
SPHK1: sphingosine kinase 1
S1P: sphingosine 1-phosphate
MMP: matrix metalloproteinase
VEGF: vascular endothelial growth factor
shTRIM14: TRIM14 short hairpin RNAs
shNC: control shRNA
HRP: horseradish peroxidase
