# Supplementary material for: TRIM14 promotes colorectal cancer cell migration and invasion through the SPHK1/STAT3 pathway
Source: Cancer Cell Int. 2018 Dec 11;18:202. doi: 10.1186/s12935-018-0701-1 (PMC6288942; doi:10.1186/s12935-018-0701-1)
Supplement: Supplementary file 1 — Additional file 1: Figure S1. TRIM14 affected the migration and invasion of CRC cells. HT-29 (A) and SW620 cells (B) were transduced with shTRIM14#1 or shNC. LoVo cells (C) were transduced with pLVX-TRIM14 or pLVX. Transwell migration and invasion assays were performed to determine the effect of TRIM14 expression on the migration and invasion of CRC cell lines at 12 h after treatment. Cells without any treatment were served as negative Control. The stained cells were counted in five random fields at 200× magnification, and the average number was taken. The average number of Control group was set as 100%, and relative migration and invasion of other groups was calculated by comparison of the cell number with the average number of control group. ***P < 0.001 versus shNC or pLVX. [file 12935_2018_701_MOESM1_ESM.docx]

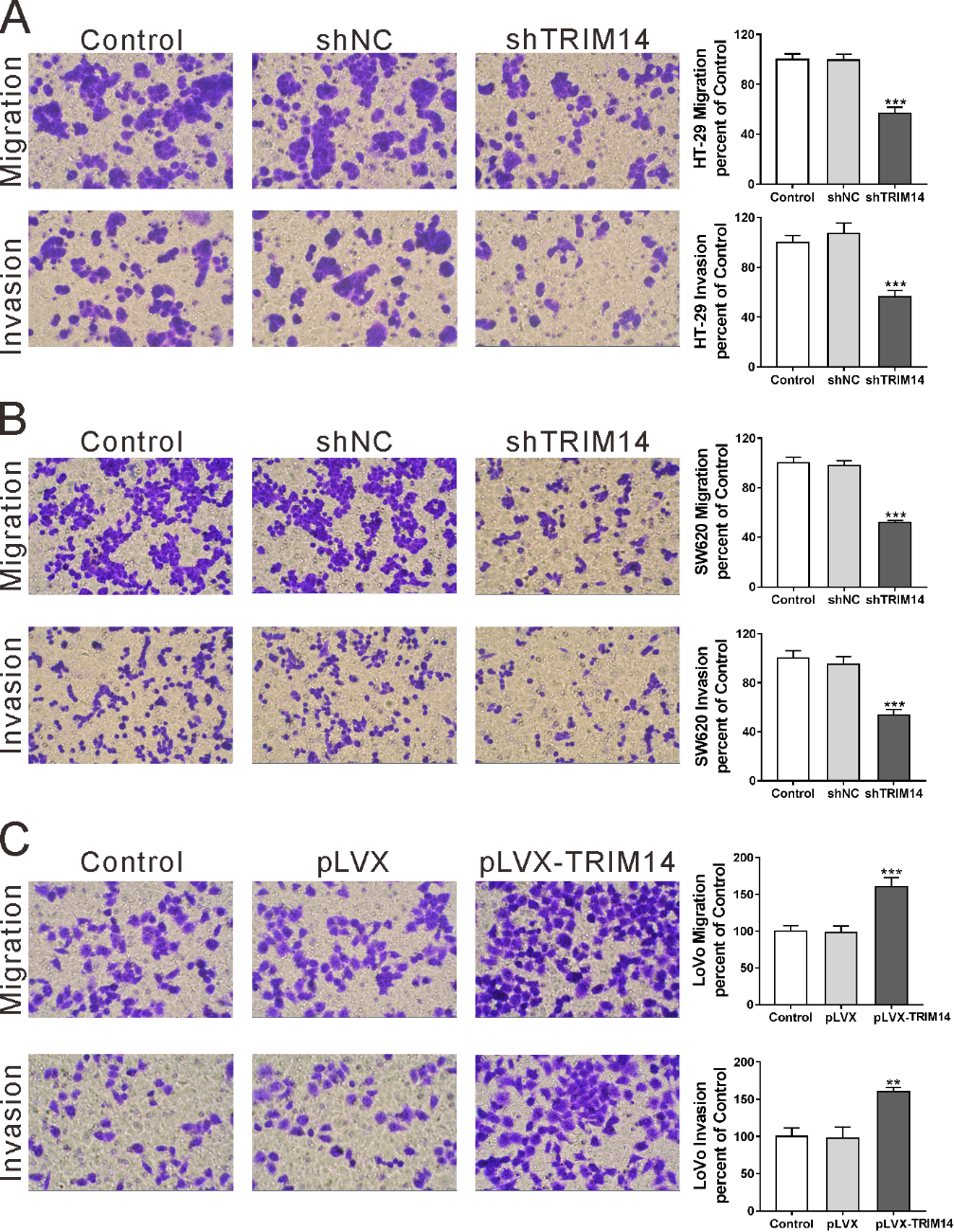


**Figure S1.** TRIM14 affected the migration and invasion of CRC cells. HT-29 (A) and SW620 cells (B) were transduced with shTRIM14#1 or shNC. LoVo cells (C) were transduced with pLVX-TRIM14 or pLVX. Transwell migration and invasion assays were performed to determine the effect of TRIM14 expression on the migration and invasion of CRC cell lines at 12 h after treatment. Cells without any treatment were served as negative Control. The stained cells were counted in five random fields at 200× magnification, and the average number was taken. The average number of Control group was set as 100%, and relative migration and invasion of other groups was calculated by comparison of the cell number with the average number of control group. ***P < 0.001 versus shNC or pLVX.
